# The scientific response to TB – the other deadly global health emergency

**DOI:** 10.5588/ijtld.21.0734

**Published:** 2022-03-01

**Authors:** R. E. Chaisson, M. Frick, P. Nahid

**Affiliations:** 1 Johns Hopkins University School of Medicine, Baltimore, MD; 2 Treatment Action Group, New York, NY; 3 UCSF Center for Tuberculosis, University of California, San Francisco, San Francisco, CA, USA

In 1993, the WHO declared TB, an airborne infectious disease, a global public health emergency and urged coordinated efforts by all nations to avert millions of deaths in the coming years.^1^ On January 30, 2020, the WHO declared COVID-19, another airborne infectious disease, a public health emergency of international concern.^2^ However, the similarity between the global responses to these two pandemics ends there. What we have witnessed in the past 2 years in terms of the scientific, public health, medical, and pharmaceutical communities to COVID-19 is nothing short of spectacular. Within 2 weeks of declaring COVID-19 a global emergency, the WHO had convened a meeting of experts and issued a research roadmap.^3^ National governments, especially that of the United States, rapidly committed vast sums of money into research at all levels, from basic virology and immunology to clinical care and prevention. Pharmaceutical companies launched development programs for new products to diagnose, treat and prevent COVID-19. As a result, diagnostics, therapeutics, and vaccines have been developed at a dizzying pace, delivering an array of tools that provide us with the means to control and end the SARS-CoV-2 pandemic. The effective and equitable deployment of those tools is a challenge of monumental proportions, but no one can claim that science has been found wanting in responding to the global crisis.

Since 1993, TB on the other hand has not been treated as a true emergency. This is perhaps because it was not new, was not escalating at a frightening pace, and had already experienced a golden age of discovery in the 20^th^ century. But even if it was not new and there were tools available to combat it, its worldwide distribution, impact on health, and mortality burden was just as dire, and the need for a rapid, coordinated, and adequately resourced scientific response was just as evident. The recently issued 2021 WHO Global Tuberculosis Report discloses the disturbing news that TB incidence remains plateaued at 10 million cases per year, but that in 2020 case detection fell by almost 20% and mortality rose for the first time in a decade to 1.5 million deaths.^4^ The decline in diagnoses and increase in mortality is directly attributable to the COVID-19 pandemic.^5^

The US National Academies of Science, Engineering, and Medicine (NASEM) held a workshop entitled “Innovations for Tackling Tuberculosis in the Time of COVID-19” in September 2021 to address challenges made even more stark by the COVID-19 pandemic, and lessons learned from the response to SARS-CoV-2.^6^ The workshop highlighted what happens when one epidemic is treated as an emergency and the other is not. With SARS-CoV-2, the development of diagnostic tests proceeded at breakneck speed: and most academic medical centers in the United States and Europe had their own inhouse PCR tests within weeks. Researchers working with government-funded consortia and industry in multiple countries launched treatment trials with remarkable alacrity – protocol development, regulatory review, institutional review board (IRB) approvals, and the launch of phase 3 randomized, registration trials for drugs ranging from hydroxychloroquine, to remdesivir, to dexamethasone took only a few weeks. The development of vaccines for COVID-19 went from genetic sequencing to phase 1 trials in less than 2 months, phase 3 in another 4 months, and Food and Drug Administration approval under an Emergency Use Authorization (EUA) within 11 months. Compared with current research on vaccines for TB, the difference is staggering (Table 1). Institutions across the board treated COVID-19 as an emergency and operated in crisis mode. The IRBs at academic health centers met multiple times per week at times to review protocols for COVID-19; the National Institutes of Health (NIH) Rapid Acceleration of Diagnostics (RADx) program issued hundreds of millions of dollars of awards in weeks, including projects focused on underserved populations (RADx-UP);^7^ additional huge investments in antivirals and vaccines were made in just a few months.

**Table 1:** Comparison of TB and COVID-19 vaccine development.

	TB^*^	COVID-19^†^
Year pathogen discovered	1882	2019
Number of vaccines licensed for use	1 (bacille Calmette-Guérin)	25
Number of vaccines in clinical trials	15	112
Development timeline of representative vaccine candidates	M72/AS01E (Gates Medical Research Institute, 2020 onward; formerly GlaxoSmithKline)	BNT162b2 (Pfizer/BioNTech)
Preclinical work begins	Early 2000s (Mtb72F, Corixa Corp)	January 2020 (BioNTech)
First Phase I trial starts	2004 (results published 2009)	May 2020 (combined phase I/II)
Pivotal Phase II trial starts	2014 (starts) 2018 (primary analysis published)	July 2020 (combined phase II/III)
Phase III starts	2023 (expected start)	
First approval	?	December 2020 (UK)

^*^ Information on TB vaccine pipeline and M72/AS01E development timeline from Treatment Action Group Tuberculosis Vaccines Pipeline Report and WHO Report of the high-level consultation on accelerating the development of the M72/AS01E tuberculosis vaccine candidate

^†^Information on COVID-19 vaccine pipeline and BNT162b2 development timeline from New York Times Coronavirus Vaccine Tracker (cited 13 December 2021).

There is no doubt that the COVID-19 pandemic is different from the TB pandemic in many ways, with its sudden appearance, rapid global spread, and widespread impact on individuals and communities. Scientists, clinicians, funders, government officials, and the general public felt (and were) personally imperiled. Despite the enormous worldwide burden of suffering and death, TB largely affects people in impoverished communities in low-income countries, and those in high-income countries feel in no danger. Nevertheless, TB remains a major killer and the pace of TB clinical research can best be described as glacial. Funding for TB research is less than half of what the United Nations and WHO estimate is necessary to achieve the End TB targets, and the Treatment Action Group’s report on research funding shows a 16-year average of just $659 million from 2005 to 2020.^8^ A comparison of investments in TB research vs. research into COVID-19 is astonishing (Table 2). But even with limited funding, there have been some triumphs in TB research in the past decade: molecular assays make diagnosis possible in less than 2 hours, rather than 2–4 weeks;^9^ treatment of multidrug-resistant TB has been shortened from 2 years of noxious, injectable agents to 6 months of an all-oral regimen;^10^ treatment of drug-susceptible TB has been shortened to 4 months, the first reduction in duration in 40 years;^11^ and treatment of TB infection has been cut from 9 months to as short as 1–3 months with safer and better tolerated regimens.^12^,^13^ However, one universal truth is that none of these transformative advances occurred as quickly as they should have.

**Table 2:** A comparison of government funding for research on TB and two estimates for COVID-19 therapeutics and vaccines, 2020

	TB^*^ US$	COVID-19^†^ US$	COVID-19^‡^ US$
Total research funding	915 million (all areas)	53 billion (vaccines only)	104 billion (vaccines + therapeutics)
Funding for vaccine research	118.6 million	53.5 billion	98.9 billion
Public funding for vaccine research	77.5 million (65%)	51.4 billion (96%)	98.9 billion (100%)
Percentage of public funding committed via advanced purchase agreements	0%	88%	98%
Philanthropic funding for vaccine research	38.7 million	85.4 million	—
Private sector funding for vaccine research	2.4 million	517.8 million	—
Multilateral funding for vaccine research	0	1.4 billion (CEPI)	—
Funding for long-term consequences of disease (post-TB lung disease^15^ and long COVID)	No estimate, but minimal	1.15 billion (US only)	—

^*^ TB funding data comes from the Treatment Action Group and Stop TB Partnership report Tuberculosis Research Funding Trends, 2005–2020^8^ which tracks research expenditures (actual disbursements) across six areas of TB research: basic science, diagnostics, drugs, vaccines, operational research/epidemiology, and infrastructure/unspecified projects.

^†^The Knowledge Network on Innovation and Access to Medicines published estimates of COVID-19 vaccine funding (disbursements and commitments) with data drawn from the Policy Cures Research COVID-19 R&D trackers and ACT-Accelerator Tracker; last updated July 8, 2021.

^‡^The kENUP Foundation published estimates of public funding for COVID-19 vaccines and therapeutics (disbursements and commitments) in the first 11 months of the pandemic in January 2021.

Compared to COVID-19 and HIV grants, NIH funding opportunities for TB biomedical research are limited and reviews of TB applications proceed according to a languid schedule. Following funding awards, the sequential and often redundant regulatory and ethical review processes at each participating institution further delay activation of the research, and therefore the results. For example, a suite of TB preventive studies funded by Unitaid and addressing WHO high-priority areas (such as interactions between TB drugs and antiretroviral drugs in pregnant women and children with HIV infection) continues to be held in a stranglehold by regulatory procedures. The WHO’s Ethics Committee, supported by overtaxed volunteer experts, can take an average of 10–12 months to review a protocol. Approval by national and local IRBs in high-burden countries can then take an additional year. The overall timeline for conducting critically important TB research is scandalously long: clinical trials for TB generally take a very long time because the endpoints are slow to accrue, but most studies are unnecessarily prolonged by painfully long administrative and regulatory review processes.

The broader problem, however, is much larger than the mechanics of individual funding agencies or regulatory bodies. First, nobody is treating TB as an actual emergency! As we have seen with COVID-19, when everyone thinks it is an emergency, people act differently, and things move rapidly. At the NASEM meeting, a South African government researcher reported waiting 6 months for approval of a minor protocol amendment to a study on lifesaving treatment for multidrug-resistant TB. We have experienced similar long delays with our trials in a number of countries. Second, the clinical and public health research infrastructure is vastly underfunded and under-supported. Much of our focus is on individual researchers who clamor (justifiedly) for more money, but the remainder of the machinery of clinical research is largely neglected. COVID-19 has demonstrated what is possible when researchers, funders, and regulatory agencies unite to confront a crisis. Game-changing trials of therapeutics and vaccines can be conducted in record time without cutting corners and compromising participant safety and scientific integrity, if everyone acts as if it is an emergency. But to do so requires a radical change in our collective mindset in addition to substantially greater human and financial resources.

Operating in crisis mode for COVID-19, TB, or any other health catastrophe is difficult to sustain. But the COVID-19 pandemic has shown us what works to accelerate progress against a global threat. First, substantial funding for priority research multiplies innovation and progress. As a starting point, governments, pharma/biotech companies, and foundations must increase investment in TB research, at least to the levels laid out in the UN High Level Meeting Report and make TB a central element in global pandemic response strategies.^14^ Moving forward, the level of ambition must be raised. There is a growing recognition from the COVID-19 experience that the funding targets for TB research are far too low – and the scale-up of newly developed tools is far too slow. Governments and other funders must commit to more to end TB by 2030. Second, the funding timeline can be greatly reduced; peer review for NIH HIV-related grants serves as a useful model for TB applications, with review occurring within 2–3 months of submission and funding within 6 months. If the rationale for implementing aggressive timelines for reviewing biomedical research in HIV and COVID-19 was the recognition and fear that these infections would rapidly spread and kill, then TB grants should likewise be reviewed rapidly. Third, the regulatory bottleneck must be cleared. Additional investment in regulatory and ethical infrastructure (including training and international coordination) is necessary to ensure that these critical requirements do not suffocate innovative research. Unnecessary regulatory reviews only add delay while providing no protection for study participants and their communities. Finally, governments must treat TB as a central element in global pandemic response strategies. The new focus on pandemic preparedness – most notably the beginning of negotiations by the WHO to create a legally binding pandemic treaty or similar mechanism – must include a commitment to end ongoing pandemics such as TB. If an annual 1.5 million deaths due to TB is not a pandemic, then what is?

The bottom line is that advances in TB diagnostics, treatments, and prevention that can translate into progress in TB elimination need to be pursued and then scaled up with the sense of urgency they deserve. If we do not behave like TB is a global health emergency, we will continue to see agonizingly slow progress in developing tools to End TB, as well as unacceptable suffering from a disease that has killed more than 20 million people in this century alone.
